# Intricate Interplay of Entwined Metabolic and Inflammatory Life-threatening Processes in Tumor Lysis Syndrome Complicating Prostate Cancer: A Systematic Review with a Single Institution Experience

**DOI:** 10.7759/cureus.7395

**Published:** 2020-03-24

**Authors:** Dawood Findakly, Jue Wang

**Affiliations:** 1 Internal Medicine, Creighton University Arizona Health Education Alliance / Valleywise Health Medical Center (formerly MIHS), Phoenix, USA; 2 Genitourinary Oncology, Creighton University School of Medicine / University of Arizona Cancer Center at Dignity Health St. Joseph’s, Phoenix, USA

**Keywords:** tumor lysis syndrome (tls), prostate cancer, metastasis, oncologic emergency, acute kidney injury (aki), cytokine storm, tumor necrosis factor-alpha, spontaneous tls (stls), treatment-related (ttls), systematic review

## Abstract

Tumor lysis syndrome (TLS) occurs in rapidly proliferating tumor cells, either spontaneously or after cytotoxic therapy. It has been well-documented in hematological diseases but is extremely rare in solid neoplasms, particularly in prostate cancer (PRCA). In the presence of risk factors, it can cause metabolic disturbances and be potentially fatal.

We searched PubMed, Medline, ScienceDirect, and Scopus for "tumor lysis syndrome" and "prostate cancer" and conducted a systematic review with a pooled analysis for the published literature and cases from our institution. Twenty-two TLS cases were identified (18 published in the literature and four cases from our institution). The patients' median age was 68 years (range 16-82), and most cases were prostate adenocarcinoma. The median prostate-specific antigen (PSA) was 374 (range 66.7-10,867). Ten cases (45.5%) had spontaneous TLS (STLS) while 12 cases (54.5%) were treatment-related (TTLS). All patients had elevated lactate dehydrogenase (LDH) with other biochemical variables, and all underwent aggressive supportive therapy. Eleven patients underwent hemodialysis, 12 patients received rasburicase, while three patients received allopurinol. The mortality rate was 75% among 12 cases of TTLS, and it was 30% of the 10 cases with STLS.

Among patients with PRCA, both TTLS and STLS linked to very high mortality. Early identification of TLS would substantially attain improved survival outcomes. Hence, physicians should consider TLS as a differential diagnosis when evaluating AKI and electrolyte abnormalities, particularly in patients with metastatic PRCA and high disease burden, even before the initiation of cytotoxic therapy.

## Introduction and background

Tumor lysis syndrome (TLS) is a major, life-threatening oncological emergency where hasty damage of tumor cells ends in a constellation of critical metabolic derangements. These constitute hyperuricemia, hyperphosphatemia, hyperkalemia, and hypocalcemia, leading to an acute kidney injury (AKI), which further worsens TLS metabolic abnormalities and outcomes [1-5]. TLS divided into spontaneous TLS (STLS) and treatment-related (TTLS). Although TLS is widely known to occur in patients with rapidly proliferating chemosensitive hematologic diseases, it can seldom happen in solid tumors. Recent papers were published indicating prostate cancer (PRCA) as being one of the solid tumors that can exceptionally rarely be complicated by TLS [6-24]. This study's objective is to investigate the clinical characteristics, management, and outcomes of TLS, a rare oncologic emergency, in patients with PRCA.

## Review

Aim of the study

This review aims to examine the available published information on the clinical characteristics, treatment, and outcomes of TLS in patients with prostate cancer.

Patients and methods

Literature Search Strategy

A systematic analysis through a comprehensive literature search was conducted and pooled with cases from our institution. We searched PubMed, Medline, ScienceDirect, and Scopus for "tumor lysis syndrome" and "prostate cancer." The identified list of cases and abstracts were reviewed and screened for any additional articles of interest from reference lists.

Data Extraction and Statistical Analysis

We extracted data on age at diagnosis, histological subtypes, prostate-specific antigen, Gleason score, diagnostic radiology, comorbid status, treatment, and outcomes were also recorded when available. Four cases from our institution were included in our analysis. We interpreted the pooled data and summarized it through descriptive statistics using central tendency and dispersion measures.

Results

We recognized 18 publications where TLS occurred in the setting of PRCA (14 case reports and four meeting abstracts). Out of the 18 cases of TLS, we identified 12 TTLS cases and six STLS cases. Furthermore, we identified four cases of STLS diagnosed in our institutional database. The final cohort was consistent with a total of 22 PRCA patients with TLS.

**Figure 1:**
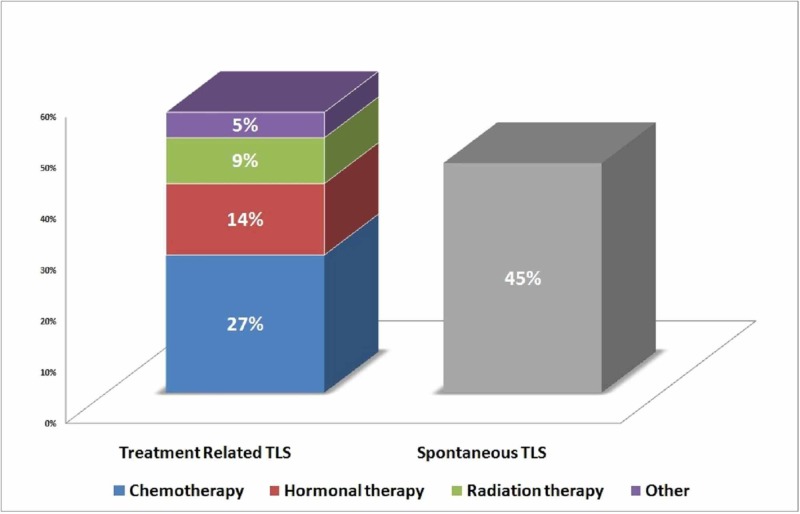
Incidence rates of treatment-related versus spontaneous TLS in PRCA patients TLS: tumor lysis syndrome; PRCA: prostate cancer.

The demographics, clinicopathologic features, and survival outcomes of 22 cases of TLS in PRCA are summarized in Table 1. The mean age (± SD) was 63.5 (±14.5) years. The median prostate-specific antigen (PSA) was 374 (range 66.7-10,867 ng/mL). Most cases were prostate adenocarcinoma (except prostate rhabdomyosarcoma in one case, prostate small cell carcinoma (SCC) in two cases, and pathology type not documented in four cases). High-grade Gleason 8+ PRCA documented in 80% of cases with available data, including two cases of prostate SCC. All cases (100%) reported distant metastases. Of the cases with available data, 100% had bone metastasis and 52% had liver metastasis. Ten cases (45.5%) had STLS. In contrast, 12 TLS cases (54.5%) were associated with a variety of treatment regimens before developing TLS; out of these, six patients received chemotherapy (27.3%), three cases received hormonal treatment (13.6%), two received radiation therapy (9.1%), and one case developed TLS after treatment with methylprednisolone for pembrolizumab-induced acute liver injury (4.5%). All patients had elevated lactate dehydrogenase (LDH) with other biochemical variables such as uric acid, potassium, phosphorus, and creatinine. All patients received aggressive supportive therapy, 11 (50%) underwent hemodialysis, 12 (54.5%) received rasburicase, and three (13.6%) received allopurinol (Figure 1).

**Table 1:** Summary of available publications and our hospital data for characteristics of patients with TLS in PRCA PRCA: prostate cancer; TLS: tumor lysis syndrome; PSA: prostate-specific antigen; Mets: metastases locations; Ref: references; ND: no data; STLS: spontaneous TLS; SCC: small cell carcinoma; LN: lymph nodes; TURBT: transurethral resection of bladder tumor

Author	Year	Age	Histology	Gleason score	PSA	Mets	Treatment preceding TLS	Time to TLS (days)	Rasburicase	Other management	Outcome	Ref
Sorscher	2004	80	Adenocarcinoma	3+3	348	Bone	Docetaxel, dexamethasone	1	No	Furosemide	Died	[6]
Tanvetyanon and Choudhury	2004	77	Adenocarcinoma	ND	10,867	Bone, liver	Flutamide, goserelin	6	No	Supportive therapy	Died	[7]
Wright et al.	2005	60	Adenocarcinoma	3+4	5520	Bone	Paclitaxel	1	No	Hemodialysis	Died	[8]
Lin et al.	2007	72	Adenocarcinoma	ND	66.7	Bone, liver	Flutamide, leuprolide, dexamethasone, medroxyprogesterone	21	No	Hemodialysis, furosemide, allopurinol	Died	[9]
Hashem et al.	2010	73	Adenocarcinoma	ND	ND	Bone	STLS	ND	No	Hemodialysis	Died	[10]
Zulqarnain et al.	2012	56	Prostate SCC	ND	6.1	Bone, LN, liver, lungs	Chemotherapy	2	Yes	Exchange transfusion for methemoglobinemia	Alive	[11]
Kaplan et al.	2012	60	Adenocarcinoma	5+4	300	Bone	Radiation therapy to shoulder	6	Yes	Sodium bicarbonate	Died	[12]
Nguyen and Ticona	2014	72	Adenocarcinoma	ND	ND	Bone, LN	STLS	ND	Yes	Supportive therapy	Alive	[13]
Watanabe and Tanaka	2014	16	Prostate rhabdomyosarcoma	ND	ND	Bone, LN	STLS	ND	Yes	Supportive therapy	Alive	[14]
Mazzoni	2016	62	Adenocarcinoma	ND	ND	Bone, LN, bladder	Radiation, TURBT, leuprolide, bicalutamide	ND	Yes	Hemodialysis, sodium bicarbonate	Died	[15]
Serling-Boyd et al.	2017	56	Adenocarcinoma	5+4	548	LN, liver	STLS	ND	Yes	Sodium bicarbonate, furosemide, allopurinol	Died	[16]
Ignaszewski and Kohlitz	2017	69	Adenocarcinoma	ND	ND	Bone, liver	STLS	ND	Yes	Hemodialysis, sodium bicarbonate	Died	[17]
Bhardwaj and Varma	2017	67	ND	ND	4500	Bone, LN, liver	Docetaxel	3	No	Supportive therapy	Died	[18]
Gongora et al.	2018	46	Adenocarcinoma	4+4	917	Bone, LN, liver, lungs	Carboplatin, etoposide	5	Yes	Supportive therapy	Alive	[19]
McGhee-Jez et al.	2018	49	Adenocarcinoma	ND	24.9	Bone	STLS	ND	No	Hemodialysis, allopurinol, prednisone	Alive	[20]
Oshima et al.	2019	77	ND	5+4	>1000	Bone, LN, liver	Cabazitaxel	3	Yes	Hemodialysis, sodium bicarbonate	Died	[21]
Sharma and Lane	2019	59	ND	ND	2.1	LN, liver, adrenal glands	Enzalutamide	30	Yes	Hemodialysis	Died	[22]
Mayer and Zarouk	2019	71	ND	ND	ND	ND on metastatic sites	Methylprednisolone for pembrolizumab-induced acute liver injury	3	Yes	Hemodialysis	Alive	[23]
Case 1, Wang	2016	72	Adenocarcinoma	4+5	746	Bone, LN	STLS	ND	No	Supportive therapy	Alive	[24]
Case 2, Wang	2018	53	Adenocarcinoma	4+4	374	Bone, LN, lungs	STLS	ND	No	Hemodialysis	Alive	[24]
Case 3	2018	82	Adenocarcinoma	ND	ND	Bone, LN, liver	STLS	ND	No	Supportive therapy	Alive	[24]
Case 4	2019	69	Prostate SCC	ND	ND	Bone, LN, liver, lungs, brain, adrenal	STLS	ND	Yes	Hemodialysis	Alive	[24]

The mortality rate was 75% among 12 cases of TTLS, with a median survival of 4.5 days (range 1-30) from diagnosis of TLS to death, and 30% in the 10 cases with STLS. There was no reported mortality in the four cases recognized and managed in our institution.

Discussion

In our cohort, several findings are noteworthy. First, the high mortality in TLS most often occurs in PRCA patients with advanced-stage metastatic disease, significant disease burden, high Gleason score, and aggressive histology. Second, TLS in PRCA, particularly TTLS, displays poorer prognosis when compared to hematologic malignancies [25-27]. Third, we recognized two patients with primary prostate SCC. Similar to SCC in other locations, prostatic SCC histology has been linked to TLS and is known for its aggressive biological behavior and rapid proliferation rate [28-29]. Molecular studies identified a germline breast cancer susceptibility 2 (BRCA2) gene mutation in both cases. BRCA-positive PRCA patients tend to have a higher Gleason score, higher PSA, higher grade of tumor proliferation, and higher rate of metastasis, predisposing them to an early-onset, aggressive, and potentially fatal disease as compared to patients with BRCA negative tumors [30-33]. 

Research in animal models proposed additional mechanisms that might explain the higher mortality of TLS. Studies in mice found disseminated microemboli from lysed tumor cells during the histopathological postmortem examination; these result in widespread tissue damage, multiorgan failure, and death [34-35]. Those findings closely mimic the human autopsy findings of disseminated tumor embolism leading to massive tissue organ necrosis and death [36-37]. Furthermore, concurrent disseminated intravascular coagulation was proposed as a probable mechanism that could synergistically lead to increased mortality in patients with TLS through common pathogenesis and pro-inflammatory cytokine-mediated processes [13-14]. Further, upon investigating this entity, cytokines, especially TNF-alfa (TNFα) and Interferon-gamma (IFNγ), are considered key mediators of what is known as a "cytokine storm," first described by Aikawa in 1996 [38]. The cytokine storm reported having a positive correlation with the tumor burden and was thought to play a vital role in the pathophysiology of TLS [39-41]. A cohort by Nakamura et al. found an elevated serum interleukin-6 (IL-6), IL-8, and IL-10 cytokine levels in all treatment-related TLS patients and reported a 100% survival rate after receiving continuous hemodiafiltration for cytokine removal [41].

We were able to identify four cases of TLS in PRCA patients managed in our 450+ bed hospital, two of which were in the last 24 months. Since our last publication on this matter, we continued to have improved outcomes through our continued efforts for early identification of TLS in patients with PRCA [24,27].

Limitations of the study

Due to the inherent nature of retrospective studies, we were not able to perform a comprehensive assessment of factors that may impact the prognosis of TLS in prostate cancer patients, including the performance status and comorbid conditions. Larger sample size would further contribute to the stratification of the risk of patients based on treatment type before TLS development. Despite the limitations, this systematic review presents the most updated real-world insight regarding the diagnosis and prognosis of TLS in patients with metastatic prostate cancer. Our study will contribute to the evolving understanding of TLS in solid neoplasms and have implications for future cancer treatment paradigms in the era of targeted therapy.

## Conclusions

Our study underscores that TLS is a real oncologic emergency, especially among patients with advanced PRCA. A heightened state of readiness orchestrated with increased attention to improve early detection of electrolytes abnormalities is immensely important for early TLS recognition and prompt intervention in patients with newly diagnosed metastatic PRCA even before starting cancer therapy. Further research is needed to determine the true incidence and molecular markers for TLS in solid tumors, which could, therefore, aid in improving outcomes.
